# Immune-Mediated Aggravation of the *Campylobacter concisus*-Induced Epithelial Barrier Dysfunction

**DOI:** 10.3390/ijms22042043

**Published:** 2021-02-19

**Authors:** Praveen Kumar Nattramilarasu, Fábia Daniela Lobo de Sá, Jörg-Dieter Schulzke, Roland Bücker

**Affiliations:** Institute of Clinical Physiology/Nutritional Medicine, Medical Department, Division of Gastroenterology, Infectiology and Rheumatology, Charité—Universitätsmedizin Berlin, 12203 Berlin, Germany; praveen-kumar.nattramilarasu@charite.de (P.K.N.); fabia.lobo-da-fonseca@charite.de (F.D.L.d.S.); joerg.schulzke@charite.de (J.-D.S.)

**Keywords:** *Campylobacter concisus*, HT-29/B6-GR/MR cells, THP-1 cells, leaky gut model, tricellular tight junction, tricellulin, occludin, claudin, tumor necrosis factor-α (TNF-α), interleukin-1β (IL-1β), interleukin-6 (IL-6)

## Abstract

*Campylobacter concisus* is a human-pathogenic bacterium of the gastrointestinal tract. This study aimed at the contribution of the mucosal immune system in the context of intestinal epithelial barrier dysfunction induced by *C. concisus*. As an experimental leaky gut model, we used in vitro co-cultures of colonic epithelial cell monolayers (HT-29/B6-GR/MR) with M1-macrophage-like THP-1 cells on the basal side. Forty-eight hours after *C. concisus* infection, the decrease in the transepithelial electrical resistance in cell monolayers was more pronounced in co-culture condition and 22 ± 2% (*p* < 0.001) higher than the monoculture condition without THP-1 cells. Concomitantly, we observed a reduction in the expression of the tight junction proteins occludin and tricellulin. We also detected a profound increase in 4 kDa FITC-dextran permeability in *C. concisus*-infected cell monolayers only in co-culture conditions. This is explained by loss of tricellulin from tricellular tight junctions (tTJs) after *C. concisus* infection. As an underlying mechanism, we observed an inflammatory response after *C. concisus* infection through pro-inflammatory cytokines (TNF-α, IL-1β, and IL-6) released from THP-1 cells in the co-culture condition. In conclusion, the activation of subepithelial immune cells exacerbates colonic epithelial barrier dysfunction by *C. concisus* through tricellulin disruption in tTJs, leading to increased antigen permeability (leaky gut concept).

## 1. Introduction

*Campylobacter concisus* is a Gram-negative epsilon-proteobacterium discovered as a pathobiont in the human oral cavity of the patients with severe gingival inflammation or periodontitis [1]. Clinical epidemiological studies detected *C. concisus* in diarrheal feces of children and immunocompromised patients [2,3]. Most importantly, one clinical study with a large cohort of diarrheal patients discovered that *C. concisus* induced prolonged diarrhea with less fever, unlike zoonotic diarrheal pathogens *Campylobacter coli* and *Campylobacter jejuni*, which cause short-term diarrhea with more often fever and increased C-Reactive Protein (CRP) levels [4]. In our previous study, we reported that *C. concisus* impaired epithelial sodium channel (ENaC) function via activation of extracellular signal-regulated kinase (ERK) and induced claudin-8-dependent barrier dysfunction, both of which contribute to sodium malabsorption and watery diarrhea [5]. The diarrheal mechanism of *C. concisus* correlates to the diarrheal pathomechanism of lymphocytic colitis, a subtype of microscopic colitis [6]. In recent conclusions from clinical studies, *C. concisus*-infected diarrheal patients were found to exhibit a higher risk for developing microscopic colitis [7,8]. Interestingly, in lymphocytic colitis patients, the compromised colonic epithelial barrier function is accompanied by a decrease in the expression of tight junction (TJ) proteins, claudin-4, -5, and -8 [9].

Tight junctions are the essential components of intercellular interfaces in the intestinal epithelia of enterocytes which regulate the barrier function. Among TJ proteins, claudins play a vital role in sealing the paracellular space between the enterocytes (fence function) [10,11]. However, claudins also act as channels for the transport of water and ions in the intestine to regulate paracellular permeability (gate function) [12,13]. In addition to claudins, occludin and tricellulin play a crucial role in regulating macromolecule permeability, and thereby act as a paracellular barrier to the luminal antigens [14,15,16]. The structure of tricellular TJ (tTJ) is unique with three pairs of TJ strands called central sealing elements [17], and its role in regulating the paracellular permeability was first observed in airway epithelia of guinea pigs [18]. Later, tricellulin was identified as an integral component of tTJ in vertebrate epithelia [19]. Surprisingly, tricellulin localization in tTJ was regulated by the bicellular tight junction (bTJ) protein occludin [20].

The mucosal immune system of the gastrointestinal tract is confronted with the antigens or microorganisms that pass the epithelial barrier. The lymphoid cells induce antigen-specific immune responses, whereas macrophages and granulocytes elicit non-specific immune responses [21]. However, in both inflammatory bowel disease (IBD) subtypes; Crohn’s Disease (CD) and ulcerative colitis (UC), the dysregulation of mucosal immune responses leads to an uncontrolled inflammation [22]. The inflammatory microenvironment in the dextran sodium sulfate (DSS)-induced colitis mouse model resulted in the accumulation of Toll-like receptor (TLR) responsive pro-inflammatory macrophages [23,24]. The lipopolysaccharide (LPS) component of the Gram-negative bacterial cell wall, which acts via TLR-4, was ascertained as an important virulence factor that aggravates the intestinal inflammation in IBD patients [25,26]. Furthermore, epithelial barrier dysfunction was ascertained in colonic epithelial cell monolayers in co-culture with M1 macrophages, the barrier dysfunction of which was induced by tumor necrosis factor-α (TNFα)-mediated deregulation of TJ proteins and epithelial apoptosis [27]. Earlier studies reported that *C. concisus* induces moderate changes in TJ expression and epithelial apoptosis, as a consequence of which intestinal epithelial barrier function is compromised [28,29].

A few clinical reports indicated the increased prevalence of *C. concisus* in the feces of inflammatory bowel disease (IBD) patients [30,31,32,33]. Hence, it becomes indispensable to investigate the link between intestinal inflammation and *C. concisus* pathogenesis, especially because increased intestinal colonization of *C. concisus* in IBD patients might result in exacerbation of inflammation and diarrhea [34,35]. *C. concisus* infection has indeed been shown to induce activation of immune cells and the release of pro-inflammatory cytokines [28]. However, the relative contribution of subepithelial immune cell activation on the overall intestinal epithelial barrier dysfunction induced by *C. concisus*, and the mechanisms of the epithelial barrier impairment and the pattern of tight junction (TJ) protein changes remained unexplored. Thus, the primary objective of this study was to elucidate this in more detail. For this purpose, we used an in vitro co-culture model of the colonic epithelial cell (HT-29/B6-GR/MR) monolayers with M1-macrophage-like THP-1 cells on the basal side [36,37], which depicts a cell model for the leaky gut situation as described in earlier studies from our group on *E. coli* and *C. jejuni* infections in the human colon [38,39]. As an important objective, we ascertained the TJ changes and apoptotic events induced by *C. concisus* in our in vitro co-culture model. Furthermore, to elucidate the mechanisms of the functional changes in the colonic epithelial barrier after *C. concisus* infection, we studied the cytokine release from M1 macrophages in the co-culture setting.

## 2. Results

### 2.1. Campylobacter concisus Aggravates the Barrier Disturbance of Intestinal Epithelial Cells in Co-Culture with Immune Cells 

**Figure 1:**
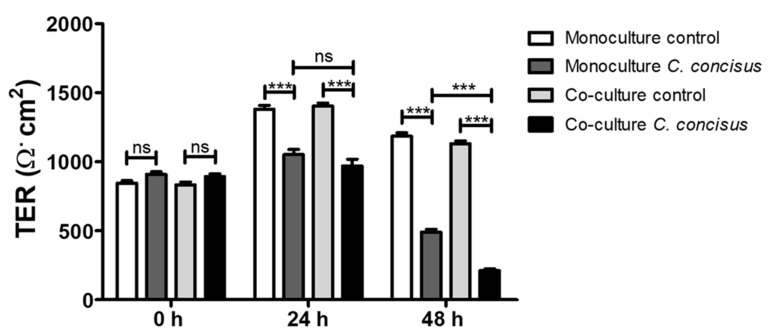
Transepithelial electrical resistance (TER) of *Campylobacter concisus*-infected colonic epithelial cell monolayers in monoculture and co-culture (with M1-macrophage-like THP-1 cells on the basal side) conditions. (A) Changes in TER in uninfected controls (white/bright grey bars) in monoculture and co-culture were recorded and compared with *C. concisus*-infections (dark grey/black bars) at 0 h (before infection), 24 h and 48 h post-*C. concisus* infection with a multiplicity of infection (MOI) of 200 (n = 23 in three independent experiments, *ns* = not significant, *** *p* < 0.001).

The changes in transepithelial electrical resistance (TER) of the cell monolayers were determined in both monoculture (HT-29/B6-GR/MR cell monolayers) and co-culture conditions (M1 macrophage-like THP-1 cells on the basal side of HT-29/B6-GR/MR cell monolayers). A clear decrease in TER of the *C. concisus*-infected cell monolayers was observed at 24 h post infection (p.i.) when compared to uninfected controls. However, at 24 h p.i., there was no significant difference between the TER of infected cell monolayers in the co-culture and the monoculture condition (Figure 1). At 48 h p.i., a further decrease in TER of the *C. concisus*-infected cell monolayers was observed in both monoculture and co-culture conditions. Interestingly, we observed a pronounced decrease in TER at 48 h, but not 24 h after *C. concisus* infection in the co-culture compared to the monoculture condition (Figure 1).

### 2.2. Protein Expression Changes of Claudins, Occludin and Tricellulin in Colonic Epithelial Cells after C. concisus Infection

**Figure 2:**
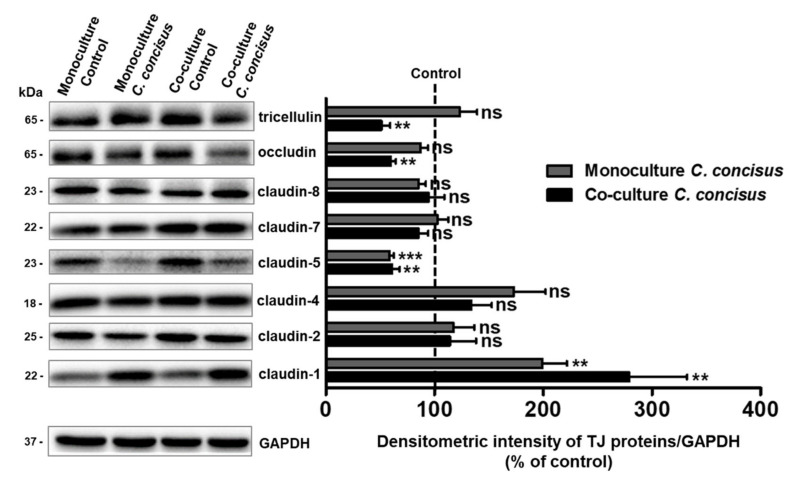
Changes in tight junction protein expression in HT-29/B6-GR/MR cell monolayers in monoculture or co-culture condition (with M1-macrophage-like THP-1 cells on the basal side) 48 h after *Campylobacter concisus* infection. Western blots and densitometry of claudins (claudin-1, -2, -4, -5, -7, -8), occludin and tricellulin (n = 6–9 in two to three independent experiments, *ns* = not significant, ** *p* < 0.01, *** *p* < 0.001).

To investigate the protein expression of tight junctions (TJs) during the TER decrease in HT-29/B6-GR/MR cells following *C. concisus* infection, a comprehensive TJ protein analysis of different claudins, occludin and tricellulin was performed in monoculture and co-culture conditions 48 h after *C. concisus* infection. Among barrier-forming claudins, we found a significant increase in claudin-1 expression and a decrease in claudin-5 expression 48 h after *C. concisus* infection, when compared to controls in both monoculture and co-culture conditions (Figure 2). The expression of claudin-2, claudin-7, and claudin-8 was unaltered after *C. concisus* infection in both monoculture and co-culture conditions (Figure 2). However, we found a tendency for an increase in claudin-4 expression in both monoculture and co-culture conditions, but this tendency failed to reach statistical significance (Figure 2). Furthermore, we observed no significant changes in the expression of occludin and tricellulin between the infected cells and controls in the monoculture condition. However, in the co-culture condition, the expression of both occludin and tricellulin decreased 48 h p.i. (Figure 2).

### 2.3. Subcellular Localization of Occludin and Tricellulin in Colonic Epithelial Cells after Campylobacter concisus Infection 

**Figure 3:**
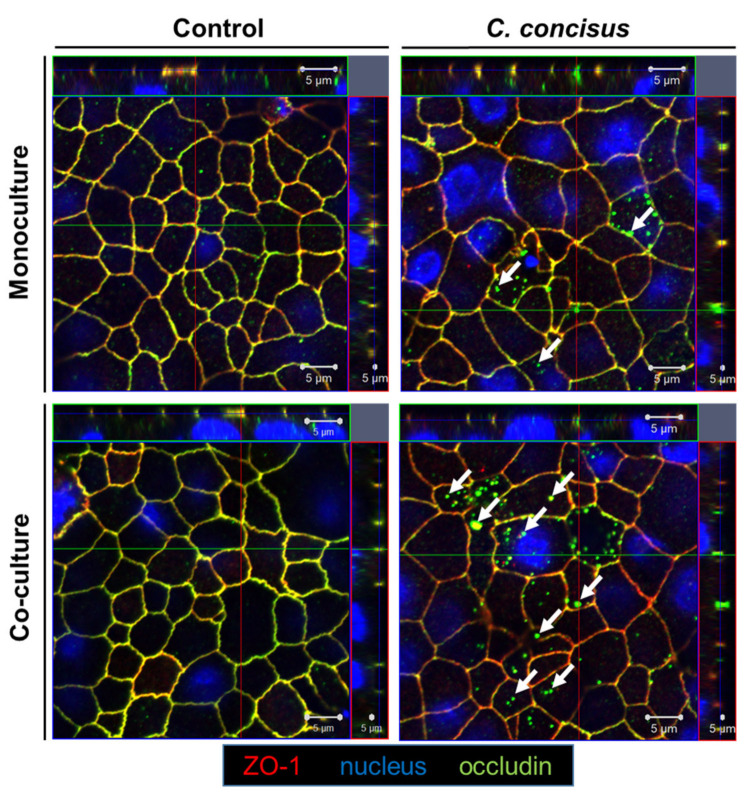
Subcellular distribution of occludin in *Campylobacter concisus*-infected HT-29/B6-GR/MR cell monolayers 48 h post infection. Occludin (green) and ZO-1 (red) are co-localized in the bicellular tight junction (bTJ) of controls in both monoculture (HT-29/B6-GR/MR cell monolayers) and co-culture conditions (HT-29/B6-GR/MR cell monolayers with M1-macrophage-like THP-1 cells on the basal side). Nuclei were stained blue with DAPI. In *C. concisus*-infected cell monolayers in monoculture and co-culture condition, white arrows indicate redistribution of occludin from bTJ into intracellular compartments.

We performed confocal laser-scanning microscopy analysis with Z-stacks of XY-scans from the epithelial cell monolayers, to determine if the delocalization of occludin and tricellulin from the intercellular junctions accompanied the protein expression changes. Forty-eight hours following *C. concisus* infection, we observed a redistribution of occludin from the bicellular tight junction (bTJ) to subapical intracellular regions in HT-29/B6-GR/MR cell monolayers maintained in co-culture condition, while zonula occludens protein-1 (ZO-1) remained intact in the bTJ. By contrast, at 48 h p.i., the redistribution of occludin from bTJ into subcellular spaces was minimal or almost absent in monoculture condition (Figure 3). In the controls of both monoculture and co-culture cell monolayers, the ZO-1 and occludin signals remained intact in the bTJ with a proper co-localization (Figure 3). 

**Figure 4:**
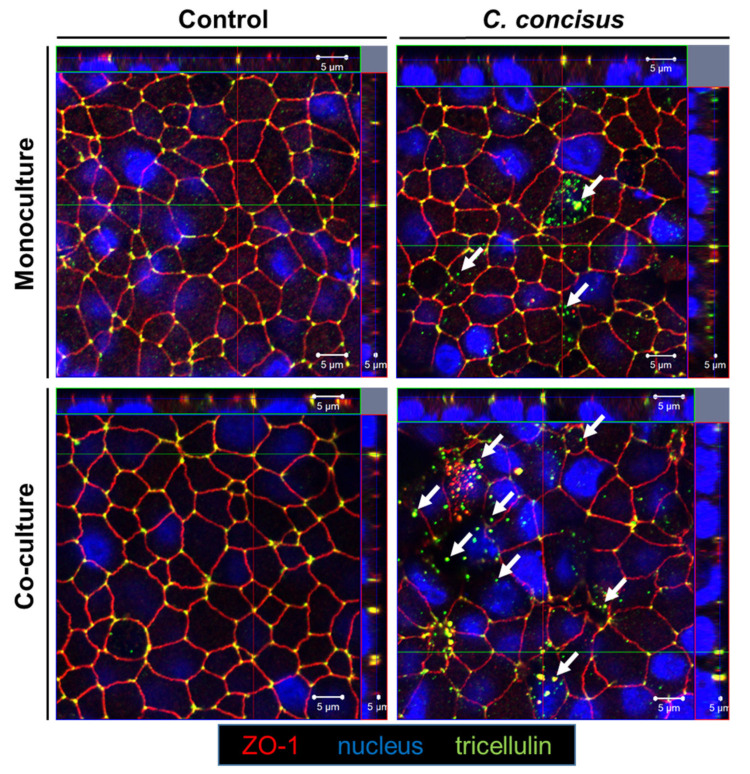
Subcellular distribution of tricellulin in *Campylobacter concisus*-infected HT-29/B6-GR/MR cell monolayers 48 h post infection. Tricellulin (green) is localized in the tricellular tight junction (tTJ) along with ZO-1 (red) in controls of both monoculture (HT-29/B6-GR/MR cell monolayers) and co-culture (HT-29/B6-GR/MR cell monolayers with M1-macrophage-like THP-1 cells on the basal side). Nuclei (blue) were stained by DAPI. In *C. concisus*-infected cell monolayers in monoculture and co-culture condition, white arrows indicate redistribution of tricellulin from the tTJ domain into intracellular compartments.

In the control cell monolayers of monoculture and co-culture conditions, tricellulin remained in the tricellular tight junction (tTJ), co-localized with ZO-1 (Figure 4). In the infected monoculture condition at 48 h p.i., redistribution of tricellulin from tTJ into intracellular compartments was rarely observed. However, in the co-culture condition together with M1-macrophage-like THP-1 cells, redistribution of tricellulin off the tTJ into intracellular regions was observed after *C. concisus* infection (Figure 4). It was also accompanied by focal leaks where ZO-1 was also redistributed off the TJ.

### 2.4. Epithelial Permeability in Campylobacter concisus-Infected Colonic Epithelial Cell Monolayers 

**Figure 5:**
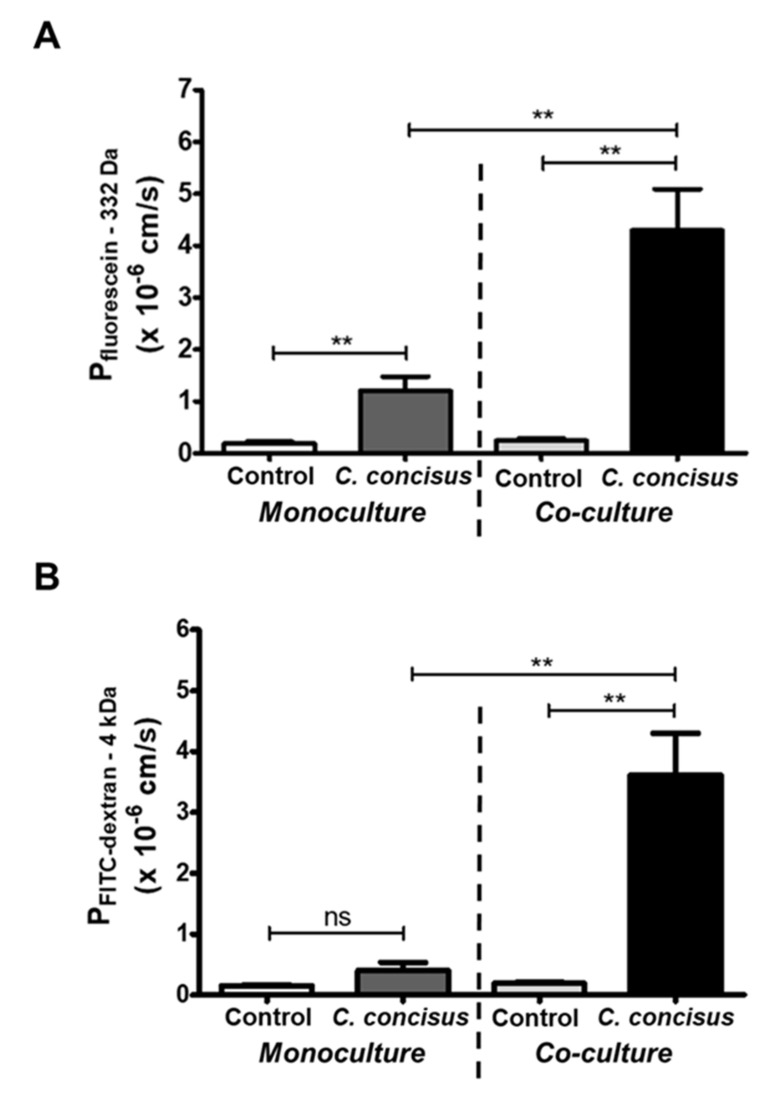
Epithelial permeability for fluorescein (332 Da) and FITC-dextran (4 kDa) in HT-29/B6-GR/MR cell monolayers under monoculture or co-culture conditions (with M1-macrophage-like THP-1 cells on the basal side) after *Campylobacter concisus* infection. (**A**) Permeability for fluorescein in monoculture and co-culture conditions 48 h after *C. concisus* infection (n = 6–8 in three independent experiments, ns = not significant, ** *p* < 0.01). (**B**) Permeability for 4 kDa FITC-dextran in monoculture or co-culture 48 h after *C. concisus* infection (n = 6–8 in three independent experiments, *ns* = not significant, ** *p* < 0.01).

We observed a pronounced drop in TER following *C. concisus* infection of cell monolayers in co-culture compared to the monoculture condition (Figure 1), representing ion permeability changes. Hence, we performed epithelial cell permeability assays for macromolecules using two fluorescent tracers of different molecular weight, namely fluorescein (332 Da) and FITC-dextran (4 kDa). As expected, we detected an increase in epithelial permeability for fluorescein (332 Da) in cell monolayers after *C. concisus* infection in both monoculture and co-culture condition (Figure 5). However, the increase in permeability for fluorescein (332 Da) in infected cell monolayers was more pronounced in the co-culture compared to the monoculture condition (Figure 5A). Furthermore, we found a significant increase in permeability to FITC-dextran (4 kDa) in *C. concisus*-infected cell monolayers under co-culture conditions, but not in the monoculture condition (Figure 5B). 

The differential pattern of the increase in permeability to macromolecules of different size indicates that the cellular structures could mediate these effects. Under monoculture conditions, *C.* *concisus*-infected cell monolayers were not permeable to FITC-dextran (4 kDa), but permeable to fluorescein (332 Da). This functional permeability pattern points to the delocalization of occludin in bTJs and/or apoptosis induction. By contrast, the increase in epithelial permeability to both fluorescein and FITC-dextran in the co-culture infection points to a loss of tricellulin in the tTJs of polarized epithelial cell monolayers (Figure 4). However, the possibility of increased apoptotic ratio should also be taken into account.

### 2.5. Epithelial Apoptosis in HT-29/B6-GR/MR Cells after Campylobacter concisus Infection 

**Figure 6:**
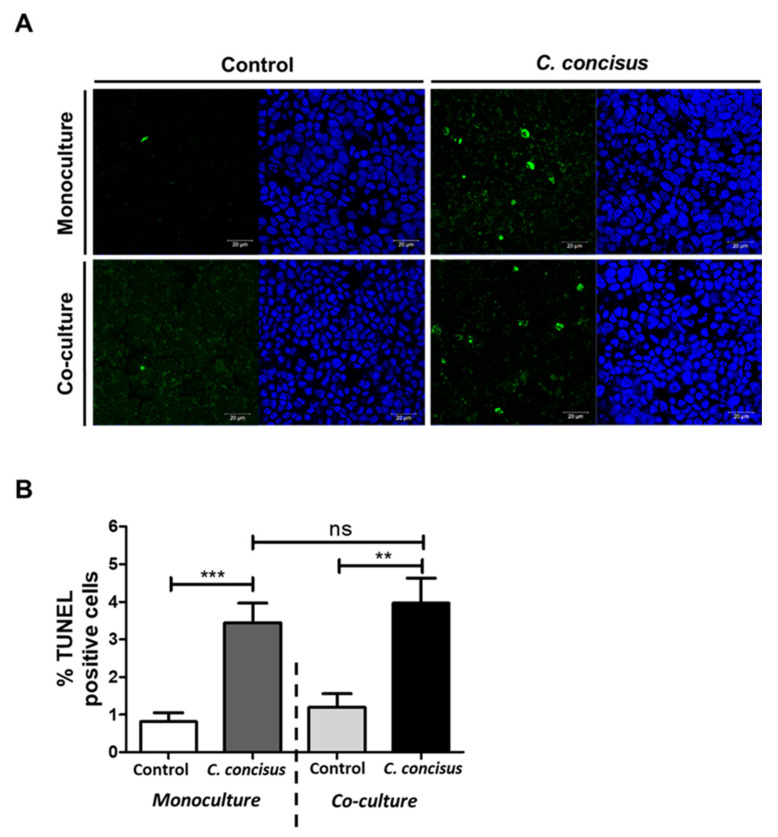
Epithelial apoptosis in HT-29/B6-GR/MR cell monolayers in monoculture or co-culture conditions (with M1-macrophage-like THP-1 cells on the basal side) after *Campylobacter concisus* infection. (**A**) The apoptotic cells in HT-29/B6-GR/MR cell monolayers were stained green by TUNEL assay kit and nuclei were stained blue by DAPI. (**B**) Percentage of apoptotic cells (TUNEL-positive) cells, quantified in five to eight randomly picked regions of the cell monolayers (n = 3 in independent experiments, *ns* = not significant, ** *p* < 0.01, *** *p* < 0.001).

We determined the extent of epithelial apoptosis in the epithelial monolayers 48 h after *C. concisus* infection. Hence, we quantified apoptosis induction with fluorescence microscopy by counting apoptotic cells in the assay of terminal deoxynucleotidyl transferase dUTP nick end labeling (TUNEL) in both monoculture and co-culture conditions. Higher numbers of apoptotic cells were detected in the infected cell monolayers in both monoculture and co-culture conditions by microscopic assessment in low-power fields (Figure 6A). 

The percentage of apoptotic cells was higher in *C. concisus*-infected cell monolayers in both monoculture and co-culture conditions when compared to controls (Figure 6B). Interestingly, there was no significant change in the apoptosis rate of the cell monolayers infected with *C. concisus* in the co-culture condition compared to the monoculture condition (Figure 6B). Apoptosis induction in the epithelium by the bacteria per se appears to be a major contributor to the barrier defect, along with the immune cell response.

### 2.6. Inflammatory Response by M1-Macrophage-Like THP-1 Cells after Campylobacter concisus Infection in the Co-Culture Setting 

**Figure 7:**
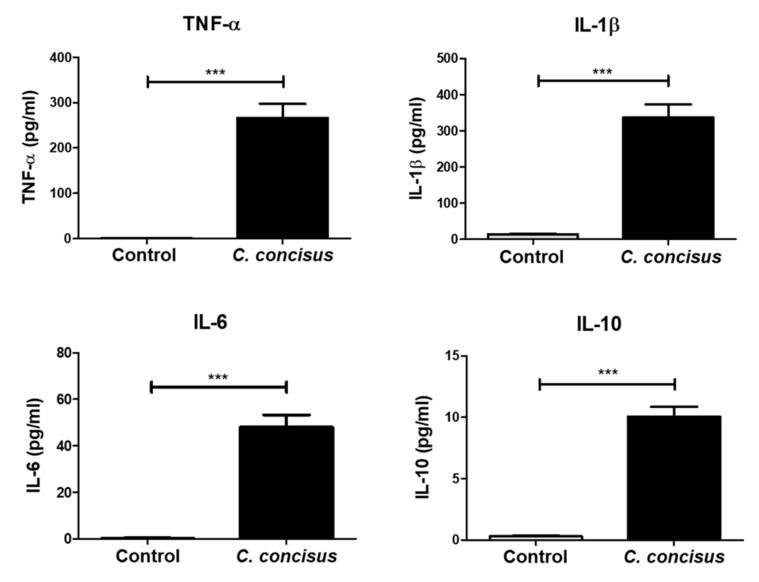
Cytokines released from M1-macrophage-like THP-1 cells in co-culture with cell monolayers. Under co-culture condition (HT-29/B6-GR/MR cell monolayers with M1-macrophage-like THP-1 cells on the basal side), the cytokines released from THP-1 cells were quantified. The cytokines TNF-α, IL-1β, IL-6, and IL-10 released from the infected cells (black bars) were compared to cytokines released from uninfected controls (bright grey bars) (n = 6 in two independent experiments, *** *p* < 0.001). The cell monolayers were infected with *C. concisus* at MOI of 200 on both apical and basal side.

To ascertain the effect of immune cells in the colonic epithelial barrier dysfunction caused by *C. concisus*, we measured the release of cytokines from the stimulated THP-1 cells 48 h after *C. concisus* infection in co-culture condition. In the culture media of the basal side of the infected cell monolayers, we assessed the release of different cytokines by Cytometric Bead Array (CBA). We found a marked increase in the release of the pro-inflammatory cytokines TNF-α, IL-1β, and IL-6, after infection, when compared to controls (Figure 7). There was also an anti-inflammatory response elicited by increased levels of IL-10 released from the THP-1 cells after *C. concisus* infection (Figure 7). Furthermore, the levels of the pro-inflammatory cytokines IFN-γ, IL-13, IL-2, and IL-17A remained unaltered (Figure S1). 

### 2.7. Cell Viability in Colonic Epithelial Cell Monolayers after C. concisus Infection 

**Figure 8:**
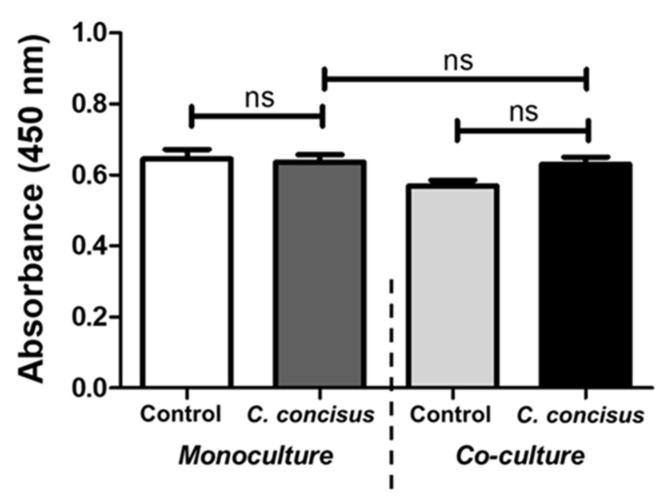
Cell viability in *Campylobacter concisus*-infected HT-29/B6-GR/MR cell monolayers. Absorbance values recorded at 450 nm with a reference wavelength of 600 nm (WST-8 in the CCK-8 assay) in controls and *C. concisus*-infected cell monolayers 48 h post infection (n = 6 in two independent experiments, *ns* = not significant). The cell monolayers in mono-culture (HT-29/B6-GR/MR) and co-culture conditions (THP-1 cells on the basal side) were infected with *C. concisus* at MOI of 200 on both apical and basal side.

We evaluated the viability of polarized epithelial cell monolayers 48 h after *C. concisus* infection. The infected HT-29/B6-GR/MR cell monolayers along with controls were subjected to the cell viability CCK-8 assay (Cell Counting Kit-8). The overall host cell viability of the *C. concisus*-infected cell monolayers in monoculture and co-culture conditions was not significantly different compared to controls (Figure 8). 

In addition to the cell monolayers, we also determined the cell proliferation rate of HT-29/B6-GR/MR cells and M1-macrophage-like THP-1 cells at 48 h p.i. with different MOI of *C. concisus* ranging from 25 to 1000. We identified that the cell proliferation rate of both HT-29/B6-GR/MR and M1-macrophage-like THP-1 cells was decreased only with an MOI of 500 and 1000 compared to controls (Supplementary Figure S2A,B). We found no significant changes in the cell proliferation rate of HT-29/B6-GR/MR cells in MOI ranging from 25 to 400 (Figure S2A). Interestingly, we also found that there was a significant increase in the cell proliferation rate of the THP-1 cells after *C. concisus* infection at an MOI of 25, 50, 100, and 200 (Figure S2B), indicating a proliferation stimulus on the immune cells by *C. concisus*. 

## 3. Discussion

As our first main result, we showed that *Campylobacter concisus* (strain AAuH 37 UC oral, an oral isolate from an UC patient in Denmark [40]) induces intestinal epithelial barrier dysfunction with a significant decrease in TER of the infected HT-29/B6-GR/MR cell monolayers at 24 h and 48 h following infection. The colonic epithelial barrier dysfunction induced by the *C. concisus* isolate confirmed its enteric pathogenic potential in the context of intestinal inflammation and diarrhea as described in other studies [34,41]. We performed the present study on barrier function only with a single strain, as our previous study revealed that several strains of *C. concisus* from healthy and diseased patients did not differ in their ability to lower intestinal epithelial barrier function regardless of the isolation sites [29].

It has also been reported that there was a significant increase in pro-inflammatory cytokines TNF-α, IL-1β, IL-12, and the chemokine IL-8 released by macrophages following *C. concisus* infection [28,41]. To date, the relative contribution and the inherent mechanisms between the subepithelial immune activation and intestinal epithelial barrier dysfunction induced by *C. concisus* have not been explored in detail. To investigate this causal link, we used an in vitro co-culture model of colonic epithelial cell monolayers with M1-macrophages, as described previously [27,36].

### 3.1. Immune-Mediated Aggravation of Campylobacter concisus-Induced Colonic Epithelial Barrier Dysfunction

Using this model, we observed a marked decrease in TER of *C. concisus*-infected cell monolayers, which was more pronounced in co-culture compared to monoculture condition at 48 h post infection. This again highlights the important role of macrophage activation and release of pro-inflammatory cytokines, TNF-α, IL-1β, and IL-6 in modulating *C. concisus*-induced colonic epithelial barrier dysfunction. This macrophage activation in our model represents a non-specific immune response. Such activation of macrophages with migration into the rectal epithelium was also observed in vivo in an ultrastructural analysis on *Campylobacter* spp.-infected patients with acute colitis [42]. Furthermore, in secondary abiotic IL-10^-/-^ mice challenged with human fecal microbiota, peroral *C. coli* infection promoted activation of macrophages in conjunction with the increased TNF-α secretion in colonic tissues 21 days post infection [43], a finding which supports the view of the immune activation being a central mechanism of the functional defects following the infection. In our present in vitro study, we also observed an activation of M1-macrophage-like THP-1 cells 48 h following *C. concisus* infection depicted through an increase in cell proliferation rate at different ranges of MOI from 25 up to 200 (Figure S2B). Interestingly, this is not unique to the infection with *C. concisus*, since in the human immunodeficiency virus (HIV) infection with compromised intestinal epithelial barrier function and leak-flux diarrhea, macrophages were also activated and elicited an inflammatory response through an increased release of the pro-inflammatory cytokines TNF-α, IL-1β, IFN-α, and IFN-γ [44]. Also, in bacterial infections of enteropathogenic *E. coli* (EPEC) [45] and *C. jejuni* [39], intestinal epithelial barrier dysfunction was accompanied by inflammatory responses.

TNF-α is a major pro-inflammatory cytokine released from the intestinal epithelium in response to infections with diarrheal pathogens like *Vibrio cholerae*, *Salmonella enterica*, and *C. jejuni* [39,46,47]. TNF-α, either alone or in combination with IFN-γ and IL-13, can regulate colonic epithelial barrier function in vitro and this mimics the differential regulation of TJ proteins observed in active IBD patients [48,49,50]. IL-1β plays a dual role as it helps to control the infection by the host-inflammatory response, and at the same time permits the entry of luminal content into the serosal compartment of the epithelium to favor inflammation [51]. Interestingly, *C. jejuni* elicited inflammatory responses through IL-1β release from THP-1 monocytes, macrophages, and inflamed colon mucosae of IL-10^-/-^ mice [36,52,53]. The inflammatory response of macrophages from *C. jejuni* infection involved TNF-α and IL-6 in addition to IL-1β to induce epithelial apoptosis affecting only claudin-1 expression in TJ of colonic epithelial cells [36]. In this study, we observed a similar inflammatory response by macrophages after *C. concisus* infection through the release of TNF-α, IL-6, and IL-1β, although in higher concentrations compared to *C. jejuni* [36,37].

In addition to the pro-inflammatory cytokines released by macrophages after *C. concisus* infection in the co-culture setting, we also detected an increase in the anti-inflammatory cytokine IL-10. This increase in IL-10 release after *C. concisus* infection could be induced by cAMP responsive element binding protein 1 (CREB1), as CREB1 upregulated mRNA expression of IL-10 after *C. concisus* infection in differentiated THP-1 macrophages, which was discerned by RNA-Seq data [54]. Furthermore, IL-10 constitutively produced by intestinal macrophages play a pivotal role in controlling excessive innate immune activation and preventing tissue damage after acute bacterial infection [55]. Beyond the immune activation promoted by *C. concisus*, the local inflammatory responses in small and large intestinal segments of IBD patients might enhance the colonization of *C. concisus* in the intestine [34,35], leading to a vicious circle of inflammation and epithelial TJ disruption. Taken together, the epithelial TJ alterations and/or apoptosis represent the molecular correlates of the pronounced intestinal epithelial barrier dysfunction resulting from macrophage activation by *C. concisus*.

### 3.2. Tight Junction Modifications in Colonic Epithelial Barrier Dysfunction Induced by Campylobacter concisus 

Although *C. concisus* was described as an emerging enteropathogen [28,29,56], sparse scientific information is available so far on TJ changes leading to intestinal epithelial barrier dysfunction. Previous studies identified that *C. concisus* induces epithelial barrier dysfunction via moderate TJ changes and epithelial apoptosis induction [28,29]. In the present study, we investigated the TJ changes in colonic epithelial cells (HT-29/B6-GR/MR) in more detail, especially under the influence of the immune activation. 

#### 3.2.1. Functional Loss of Occludin in Colonic Epithelial Cells in Co-culture with Macrophages after *Campylobacter concisus* Infection 

The expression of occludin was downregulated in membrane fractions of Caco-2 cells 48 h after *C. concisus* infection, whereas the expression of total occludin remained unaltered in a previous study [28]. In the present study, we also found that the expression of total occludin remained unaltered in HT-29/B6-GR/MR cell monolayers at 48 h after *C. concisus* infection in monoculture condition. However, under the co-culture condition with immune activation, we also ascertained a downregulation in the protein expression of total occludin after *C. concisus* infection. In addition, we detected a subcellular redistribution of occludin from the bTJ in HT-29/B6-GR/MR cell monolayers after *C. concisus* infection in co-culture condition. The reduction in occludin expression and its redistribution from bTJ following *C. concisus* infection might also result from an increased release of IL-6 and IL-1β by M1-macrophages, as reported elsewhere [57,58,59]. In addition to IL-6 and IL-1β, earlier studies reported TNF-α-induced epithelial barrier dysfunction in rat colon via downregulation of occludin and E-cadherin [60] and in DSS-induced colitis in mice [61]. To summarize, it is reasonable to conclude that the inflammatory response of M1-macrophage-like THP-1 cells facilitates downregulation of occludin expression and its dissociation from bTJ in colonic epithelial cells after *C. concisus* infection. Furthermore, occludin knock-down in MDCK cell monolayers revealed that occludin regulates paracellular permeability, with an increase in the paracellular flux of macromolecules up to 40 kDa in the absence of occludin [15]. Hence, the downregulation of occludin and its dissociation from the bTJ in HT-29/B6-GR/MR cell monolayers could be one contributing factor for an increase in epithelial permeability to small (fluorescein, 332 Da) and large (FITC-dextran, 4 kDa) macromolecules in infected cell monolayers in co-culture condition compared to monoculture condition.

#### 3.2.2. *Campylobacter concisus* Induces Tricellulin Downregulation and Redistribution from Tricellular Tight Junctions of Colonic Epithelial Cells in Co-culture with Macrophages

Tricellulin is an integral component of the intestinal epithelium regulating barrier function for macromolecules [19,20]. In co-culture condition, we observed a reduction in the protein expression of tricellulin and a profound subcellular redistribution 48 h after *C. concisus* infection. The tTJ was first identified as a bacterial target in MDCK cell monolayers, where tricellulin knock-down prevented the intercellular movement and shedding of *Shigella*, which revealed tricellulin as a receptor for bacterial invasion [62]. Direct interaction of *C. concisus* with the tTJ, as shown for *Shigella*, is possible and should be addressed in future investigations. Furthermore, in intestinal epithelial barrier damage by EPEC, tricellulin expression was reduced and a random re-distribution of tricellulin off tTJ was observed in Caco-2 cell monolayers at 4 h p.i. [63]. We identified for the first time that *Campylobacter* spp. infection downregulates tricellulin expression under the influence of immune activation to promote barrier dysfunction in the intestinal epithelial cells. Even in the absence of an inflammatory response, as present in monoculture cell monolayers, we observed a redistribution of tricellulin from the tTJ in a subpopulation of cells after *C. concisus* infection. However, a profound general redistribution of tricellulin from the tTJ was observed 48 h after *C. concisus* infection only under the influence of the inflammatory response from macrophages. In contrast to *C. concisus*, tricellulin expression remained unaltered in *C. jejuni* infections in our co-culture model of colonic epithelial cells with M1 macrophages, where inhibition of epithelial apoptosis induction attenuated the barrier dysfunction induced by *C. jejuni* [37]. However, tricellulin expression in the co-culture model was investigated only at an early time point of 22 h after *C. jejuni* infection. Investigations of tricellulin expression 48 h after *C. jejuni* infection in co-culture condition might have a different outcome. EpSG1, an effector molecule of type-III secretion system (T3SS) in EPEC, was found to exhibit a selective role in the downregulation of tricellulin via disruption of microtubular filaments [63]. Since a number of clinical isolates of *C. concisus* possess type-VI secretion system (T6SS) genes, which code for a complete T6SS apparatus [64,65], future studies should investigate the role of T6SS-dependent effector molecules in intestinal epithelial barrier dysfunction induced by *C. concisus* with respect to tricellulin downregulation.

In our present study, the loss of tricellulin from the tTJ was accompanied by a nine-fold increase in the permeability to FITC-dextran (4 kDa) in *C. concisus*-infected colonic epithelial cells in co-culture compared to monoculture condition. This correlates with the disease pathomechanisms in the colon mucosae of UC patients [66] and ileal biopsies of monozygotic twins with CD [67], where tricellulin downregulation was accompanied by an increase in paracellular permeability to 4 kDa FITC-dextran. This allows an influx of larger macromolecules and bacterial antigens into the intestinal mucosa to promote inflammatory processes. In UC patients, IL-13 was determined as the pro-inflammatory cytokine which induced tricellulin downregulation and its redistribution from the tTJ [66]. However, in *C. concisus* infection in our co-culture setting, IL-13 release from M1 macrophages was not significantly altered compared to controls (Figure S1). Hence, *C. concisus* has to be assumed to promote an IL-13-independent tricellulin downregulation and redistribution from the tTJ in colonic epithelium, e.g., through macrophage-derived TNF-α, a mechanism similar to the compromised epithelial barrier function in the duodenal mucosa of Whipple’s Disease patients [68]. Thus, with immune cells that were capable of secreting IL-13 in other macrophage models like PBMCs or in the in vivo experiments, the barrier dysfunction via tricellulin dysregulation could be even more pronounced. Tricellulin loss in the tTJ after *C. concisus* infection in our co-culture experiments could be attributed to the downregulation and redistribution of occludin from the bTJ, as loss of occludin notably affects the localization of tricellulin in the tTJ [15,20]. However, the molecular mechanism that governs the tricellulin downregulation or its redistribution resulting from *C. concisus* infection in co-culture condition has to be elucidated in future studies.

#### 3.2.3. The Role of Claudins in *Campylobacter concisus*-Induced Colonic Epithelial Barrier Dysfunction 

As a structural correlate of the epithelial barrier dysfunction induced by *C. concisus* in HT-29/B6 cells, a previous study identified a reduction in protein and mRNA expression of claudin-5 [29]. It was accompanied by an increase in epithelial permeability to 332 Da fluorescein, but not to 4 kDa FITC-dextran [29]. In our present investigation, we observed a decrease in claudin-5 protein expression 48 h after *C. concisus* infection in both monoculture and co-culture conditions. It points to a mechanism of TNF-α-dependent decrease in claudin-5 in the colon mucosae of lymphocytic colitis patients [9], as TNF-α release was evident in colonic epithelial HT-29 cells following *C. concisus* infection [28]. However, in monoculture condition, *C. concisus*-infected cell monolayers showed a 6.6-fold increase in epithelial permeability to fluorescein (332 Da). Hence, the reduction in claudin-5 expression by *C. concisus* could also influence the increase in epithelial permeability to the small macromolecule (fluorescein, 332 Da). 

We detected a decrease in the protein and mRNA expression of claudin-8 in HT-29/B6-GR/MR cell monolayers 48 h after *C. concisus* infection in our previous study, in which regulation of claudin-8 expression was influenced by stimulation with dexamethasone (D), butyrate-sodium salt (B), and aldosterone (A) [5]. In our present study, claudin-8 protein expression remained unaltered 48 h after *C. concisus* infection in HT-29/B6-GR/MR cell monolayers, presumably because they were not pre-stimulated with DBA. Claudin-1 expression was increased 48 h after *C. concisus* infection in HT-29/B6-GR/MR cell monolayers in both monoculture and co-culture conditions and this resembled the increase in claudin-1 expression 48 h following *C. fetus* and *C. coli* infections [69]. This phenomenon of an increase in claudin-1 in spite of a decrease in TER is called claudin-1 paradox. It is due to a redistribution of the (increased) claudin-1 off the tight junction domain of the epithelial cells as shown in the *C. jejuni* infection in the colonic epithelium [37,38]. The reduction in TER and upregulation of claudin-1 expression results from the pro-inflammatory cytokine TNF-α via NF-κB [70]. In contrast to the increase in claudin-1 expression after *C. concisus* infection in colonic epithelium, a recent study reported claudin-1 downregulation with a concomitant barrier dysfunction and an increase in inflammation in atopic dermatitis [71]. However, in the context of intestinal inflammation, claudin-1 expression was increased in the colon mucosae of IBD patients [72]. Furthermore, an increase in claudin-1 expression in the intestinal epithelium was also implicated in inflammation-induced colon tumorigenesis [73]. However, it is unknown whether claudin-1 has self-regulatory properties in this respect or has been subject to expression regulation regardless of the inflammation. At this point, we can speculate that the claudin-1 redistribution off the tight junction domain into intracellular compartments in the colon induces a counter-regulation with claudin-1 transcription upregulation to maintain barrier function. 

### 3.3. Epithelial Apoptosis in the Regulation of Intestinal Epithelial Barrier Dysfunction Induced by Campylobacter concisus 

Epithelial apoptosis was identified as a major determinant in the colonic epithelial barrier dysfunction induced by *C. concisus* in HT-29 cells at 48 h p.i., which supports the concept of dysfunction through an increase in the paracellular flux of ions and small macromolecule (fluorescein, 332 Da), but not large macromolecule (FITC-dextran, 4 kDa) [29]. This leak-flux pathomechanism in intestinal epithelium with up-regulated epithelial apoptoses was also detected in duodenal biopsies of patients with *Giardia lamblia* infection [74]. Furthermore, this was evident in *C. fetus* infection in HT-29 cells. However, *C. fetus* promoted an increase in permeability to 10 kDa FITC-dextran through focal leaks in cell monolayers [68]. In a recent study with a co-culture model of colonic epithelial cells with macrophages, *C. jejuni* was also found to induce epithelial apoptosis in the colonic epithelium with an increase in paracellular flux for 4 kDa FITC-dextran at 22 h p.i. [37]. Therefore, we aimed to determine if epithelial apoptosis contributes to the pronounced colonic epithelial barrier dysfunction after *C. concisus* infection in our co-culture model.

There were no significant changes in the number of apoptotic cells between the cell monolayers infected in monoculture and co-culture conditions, although we found a significant increase in the paracellular flux of 4 kDa FITC-dextran in co-culture infected cell monolayers compared to monoculture infected monolayers. This suggests that an increase in large macromolecule (FITC-dextran, 4 kDa) flux was caused by the disruption of tricellulin in the tTJ, but not by epithelial apoptosis. However, the epithelial apoptotic rate was significantly higher in infected cell monolayers compared to controls in both monoculture and co-culture conditions. Hence, this could be one contributing factor along with dysfunctional occludin and claudin-5 to influence the small macromolecule (fluorescein, 332 Da) flux across the colonic epithelium. Most importantly, the necrotic cell death mechanisms did not influence the barrier dysfunction of *C. concisus*-infected HT-29/B6-GR/MR cell monolayers, as no significant differences were observed in cell cytotoxicity between the controls and infected monolayers (Figure 8). As a piece of supportive evidence, we also show that the proliferation of HT-29/B6-GR/MR cells in 96-well plates was not significantly altered at 48 h p.i. with respect to controls up to an MOI of 400 (Figure S2A).

Taken together, *C. concisus* induce epithelial barrier dysfunction in colonic epithelium through epithelial apoptosis and claudin-5 downregulation in the absence of inflammatory response from macrophages. *C. concisus* infection also facilitates flux of ions and smaller macromolecules like fluorescein. It confirms the concept of a leak-flux pathomechanism exhibited by *C. concisus*, as proposed in previous studies. Most importantly, with the macrophage activation and cytokine induction, which resembles the in vivo situation of the inflamed colon in campylobacteriosis, the colonic epithelial barrier damage promoted by *C. concisus* exacerbates via occludin and tricellulin disruption. Tricellulin loss in the tTJ could create a large channel in the central tube of the epithelial tricellular contacts in the intestine. It allows the influx of large macromolecules, for instance—bacterial toxins through the tTJ, and thereby exacerbates intestinal inflammation in campylobacteriosis or IBD patients leading to watery or bloody diarrhea.

## 4. Materials and Methods

### 4.1. Cell Culture

We used HT-29/B6-GR/MR cell line (classical HT-29/B6 cell line, stably transfected with glucocorticoid and mineralocorticoid-receptors) to analyze the epithelial barrier function following *C. concisus* infection. HT-29/B6-GR/MR cells were cultured in Roswell Park Memorial Institute (RPMI) media (Sigma Aldrich, St. Louis, MO, USA) for one week at 37 °C in a humidified atmosphere (95% air/5% CO_2_). RPMI media were supplemented with 10% fetal calf serum (FCS; Gibco, Carlsbad, CA, USA), 1% penicillin/streptomycin (Gibco, Carlsbad, CA, USA), 500 IU/mL G418 (Merck Millipore, Billerica, MA, USA), and 200 μg/mL hygromycin B (Biochrom GmbH, Berlin, Germany). The human monocyte leukemia cell line THP-1 (ATCC TIB-202) was provided by Dr. Verena Moos, Medical Department, Division of Gastroenterology, Infectiology, and Rheumatology, Charité–Universitätsmedizin Berlin, Berlin, Germany. The THP-1 monocytes were grown as suspended cells for seven days in RPMI media supplemented with heat-inactivated 10% FCS and 1% penicillin/streptomycin. The cells were then subjected to centrifugation at 130 g for 10 min at 22 °C. The cell pellets were re-suspended in RPMI media supplemented with heat-inactivated 10% FCS without any antibiotic supplements. The cell count was determined and 1.8 × 10^5^ cells were seeded into individual compartments of 12-well plates (Falcon Polystyrene Microplates, Thermo Scientific, Waltham, MA, USA) and treated with 100 nM phorbol 12-myristate 13-acetate (PMA; Sigma Aldrich St. Louis, MO, USA) for 24 h to differentiate them into M1 type macrophages. 

**Figure 9:**
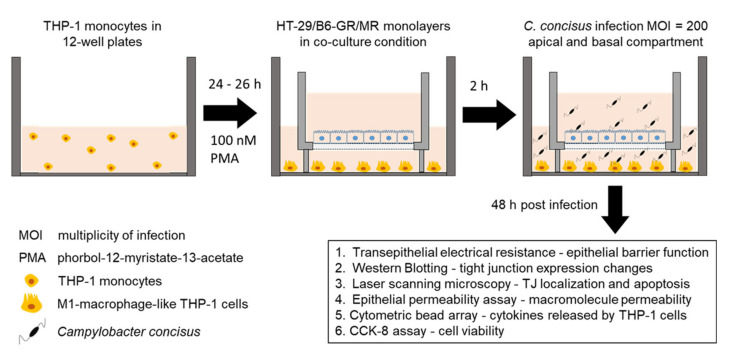
Leaky gut cell model to investigate colonic epithelial barrier dysfunction induced by *Campylobacter concisus*.

The confluent HT-29/B6-GR/MR cells in a culture flask were trypsinized and seeded on Millicell PCF filters of 3 μm pore size (Merck Millipore, Billerica, MA, USA). Seven to nine days old HT-29/B6-GR/MR cell monolayers were washed and incubated overnight in RPMI media supplemented with heat-inactivated 10% FCS without any antibiotic supplements. In parallel, M1-macrophages differentiated from THP-1 monocytes were washed and incubated in RPMI media with heat-inactivated 10% FCS without any antibiotic supplements. After overnight incubation with antibiotic-free medium, the HT-29/B6-GR/MR cell monolayers on 3 µm PCF filters were placed over the single wells of 12-well plates containing M1-macrophage-like THP-1 cells (Figure 9). This depicts the leaky gut in vitro co-culture model of colonic epithelial cells with M1-macrophages on the basal side as described in a previous study [36]. In parallel, we also used monoculture HT-29/B6-GR/MR cell monolayers in 12-well plates without THP-1 cells.

### 4.2. Transepithelial Electrical Resistance Measurements and Campylobacter concisus Infection

The transepithelial electrical resistance (TER) was recorded with chop-stick electrodes (STX2, World Precision Instruments, Sarasota, FL, USA) and a volt-ohm meter (Institute of Clinical Physiology, Charité–Universitätsmedizin, Berlin, Germany) in both monoculture and co-culture (with M1-macrophage-like THP-1 cells on the basal side) cell monolayers under sterile conditions. The cell monolayers were infected with *C. concisus* (*C. concisus* AAuH 37 UC oral [40]) on the apical and basal sides of the cell monolayers at a multiplicity of infection (MOI) of 200 (Figure 9). After infection, the cell monolayers were incubated in a microaerophilic and H_2_-containing atmospheric condition at 37 °C, as described in our previous study [5]. TER was recorded at 24 h and 48 h p.i.. The oral isolate of *C. concisus* from a UC patient (*C. concisus* AAuH 37 UC oral) was provided by Dr. Hans Nielsen, Department of Clinical Microbiology/Clinical Medicine, Aalborg University Hospital/Aalborg University, Aalborg, Denmark. 

### 4.3. Western Blotting to Determine the Expression of Different Tight Junction Proteins

The controls and infected cell monolayers in both monoculture and co-culture conditions were washed twice with PBS. The cells were then scraped out carefully from the cell monolayers and subjected to lysis for one hour in ice-cold condition with whole-cell lysis buffer (150 mM NaCl, 10 mM Tris buffer pH of 7.5, 0.5% Triton X-100, and 0.1% SDS). After lysis, the cells were centrifuged at 13,000 rpm for 30 min at 4 °C and the supernatants were collected in separate tubes. The supernatants were then subjected to sonication with 15 pulses at brief intervals of 3 s for every 5 pulses. After sonication, the cells were again centrifuged for 13,000 rpm for 30 min at 4 °C. The supernatants were then transferred to fresh vials and maintained in ice-cold conditions. The concentration of the proteins was estimated by Pierce bicinchoninic acid (BCA) assay (Thermo Scientific, Waltham, MA, USA) according to the manufacturer’s instruction. Protein samples were resolved using 10% SDS-PAGE gels and 12.5% SDS-PAGE gels, and 15 µg of proteins were used from each sample. The resolved proteins were electro-transferred to PVDF nitrocellulose membranes (Thermo Scientific, Waltham, MA, USA) with Wet/Tank Blotting system (Bio-Rad Laboratories, Inc., Hercules, CA, USA) according to the manufacturer’s instruction.

PVDF nitrocellulose membranes were subjected to incubation shaking with a blocking solution containing 1% polyvinylpyrrolidone (PVP-40; Sigma Aldrich, St. Louis, MO, USA) in tris-buffered saline (TBS) supplemented with 0.05% Tween-20 at room temperature (RT) for 1 h to avoid unspecific protein signals. Following this, we incubated the membranes with primary antibodies rabbit (Rb) anti-claudin-1, -2, -5, -7, and -8 (Invitrogen, Carlsbad, CA, USA), mouse (M)-anti-claudin-4 (Invitrogen, Carlsbad, CA, USA), Rb-anti-occludin (Sigma Aldrich, St. Louis, MO, USA), Rb anti-tricellulin (Invitrogen, Carlsbad, CA, USA), M-anti-β-actin (Sigma Aldrich, St. Louis, MO, USA), and M-anti-GAPDH (Merck KGaA, Darmstadt, Germany) overnight at 4 °C. Membranes were then incubated with appropriate secondary antibodies (peroxidase-conjugated goat anti-Rb and goat-anti-M, Jackson ImmunoResearch, Ely, UK) at RT for 2 h. The membranes were then detected for the bands of specific proteins with a chemiluminescence solution (Thermo Scientific, Waltham, MA, USA) using the FUSION FX7 system (Vilber Lourmat Deutschland GmbH, Eberhardzell, Germany). Protein bands were quantified by ImageJ software (Rasband, W. S., ImageJ, National Institute of Health (NIH), Bethesda, MD, USA). Densitometric analysis of the Western blots was performed by normalizing the band intensity of TJ proteins to their respective β-actin or GAPDH band intensities.

### 4.4. Immunofluorescence Staining and Confocal Laser-Scanning Microscopy to Determine Tight Junction Protein Localization

Forty-eight hours after *C. concisus* infection, HT-29/B6-GR/MR cell monolayers under monoculture and co-culture conditions were analyzed for TJ localization along with the controls. The cell monolayers in 3 μm PCF filters were fixed using 2% paraformaldehyde (PFA; Electron Microscopy Sciences, Hatfield, PA, USA) at RT for 20 min. After fixation, the cells were quenched with 25 mM Glycine (Biomol GmBH, Hamburg, Germany). The fixed cells were washed twice with PBS (with Ca^2+^/Mg^2+^) and permeabilized with 0.5% Triton X-100 (Sigma Aldrich, St. Louis Missouri, MO, USA) for 7 min at RT. The permeabilized cells were washed and incubated with blocking solution (1% (*v/v*) goat serum (Gibco, Carlsbad, CA, USA)) diluted in PBS (with Ca^2+^/Mg^2+^) at RT for 30 min. The primary antibodies, Rb-anti-occludin (Thermo Scientific, Waltham, MA, USA), Rb-anti-Tricellulin (Invitrogen, Carlsbad, CA, USA), and M-anti-human ZO-1 (BD Biosciences, Franklin Lakes, New Jersey, USA) were diluted with the blocking solution to optimal concentrations. After blocking, the cells were incubated with primary antibodies at 37 °C for 45 min. Then, the cell monolayers were incubated with secondary antibodies, goat anti-Rb green, Alexa Fluor Plus 488 nm and goat-anti-M red, Alexa Fluor 594 nm (Invitrogen Carlsbad, CA, USA) for 45 min at 37 °C. Following this, the cell monolayers’ nuclei were stained with 4′-6-diamidino-2-phenylindole dihydrochloride (DAPI; Roche AG, Basel, Switzerland) at a dilution of 1:1000 in blocking solution. Later, the cell monolayers were dried and mounted on a glass slide using the mounting solution ProTaq Mount Fluor (Biocyc, Luckenwalde, Germany) and fixed with coverslips. The control and the infected cell monolayers were assessed for localization and redistribution of TJ proteins occludin and tricellulin, co-stained with zonula occludens protein-1 (ZO-1) using confocal laser-scanning microscopy (CLSM, Zeiss LSM 780, Jena, Germany). The individual Z-stacks of the cell monolayers were recorded using the laser scan function.

### 4.5. Epithelial Permeability Assay

Forty-eight hours after *C. concisus* infection, the HT-29/B6-GR/MR cell monolayers along with controls were incubated with RPMI medium supplemented with 50 µg/mL gentamycin for 2 h at 37 °C. After incubation with gentamycin-supplemented RPMI media, the fluorescent markers of different molecular size, fluorescein (332 Da; 100 µM) and FITC-dextran (4 kDa; 200 µM) were used to measure the paracellular permeability of the epithelial cell monolayers. The permeability of the cell monolayers was measured through unidirectional flux for fluorescein and FITC-dextran in 12-well plates at 37 °C over 1–2 h. In fluorescein flux measurements, the samples from the basolateral side were obtained every 15 min up to 1 h. For FITC-dextran, the samples were obtained every 30 min up to 2 h. The fluorescence signals were measured in a spectrophotometer (Tecan GmbH, Maennedorf, Switzerland) at optimal wavelengths to calculate the flux of fluorescein and FITC-dextran. The permeability was then calculated from flux over concentration difference. 

### 4.6. Quantification of Epithelial Apoptosis

To detect epithelial apoptosis in *C. concisus*-infected colonic epithelial cell monolayers, we used TUNEL assay kit (In situ Cell Death Detection Kit, Roche AG, Mannheim, Germany). We stained the cell monolayers with the enzyme solution, terminal deoxynucleotidyl transferase (TdT) according to the manufacturer’s instructions. Nuclei were then stained by DAPI (Roche AG, Basel, Switzerland) at the optimum concentration. The cellular apoptosis was visualized using confocal laser-scanning microscopy (CLSM; Zeiss LSM 780, Jena, Germany). The apoptotic cells were then counted in randomly picked regions, with 5 to 8 regions in controls and infected cell monolayers at a low power field of approximately 150 to 200 cells. Then, the percentage of TUNEL-positive cells was estimated from the ratio of apoptotic or TUNEL-positive cells to the total number of cells in the regions picked for quantification. 

### 4.7. Cytometric Bead Array to Measure the Cytokine Release 

We measured the pro-inflammatory cytokines released from M1-macrophage-like THP-1 cells 48 h after *C. concisus* infection in co-culture cell monolayers. The media from the basal compartment of the *C. concisus*-infected and control cell monolayers were collected from 12 well plates. This was subjected to analysis of multiple cytokines (IL-1β, IL-13, IL-4, IL-6, IL-10, IL-17A, IFN-γ, and TNF-α) with capture bead technology of human cytometric bead array kit (CBA; BD Biosciences Human Th1/ Th2/Th17 Kit and Flex Set IL-13 and IL-1β, Franklin Lakes, NJ, USA) according to the manufacturer’s protocol. The cytokines were then measured with flow cytometry by Fluorescence-activated Cell Sorting (FACS) Canto II (BD Biosciences; Franklin Lakes, NJ, USA). The data from the cytokine measurements were analyzed with FACP Array software v3.0 (BD Biosciences, Franklin Lakes, NJ, USA). 

### 4.8. Cell Viability Assay

The cell proliferation rate of HT-29/B6-GR/MR cell monolayers 48 h after *C. concisus* infection (MOI = 200, on apical and the basal side) were evaluated by CCK-8 assay (Cell Counting Kit-8, Thermo Scientific, Waltham, MA, USA). In addition to this, we also determined the individual cell proliferation rate of HT-29/B6-GR/MR cells and M1-macrophage-like THP-1 cells in 96-well plates. HT-29/B6-GR/MR cells were seeded into 96-well plates and incubated overnight at 37 °C in a humidified atmosphere (95% air/5% CO_2_). The cells were washed and incubated overnight with antibiotic-free RMPI medium. In parallel, THP-1 monocytes were differentiated into M1 macrophages with PMA for 24 h. Following this, M1 macrophages were also washed and incubated overnight with antibiotic-free RPMI medium. Then, both HT-29/B6-GR/MR and THP-1 cells were infected with *C. concisus* at MOI of 25, 50, 100, 200, 400, 500, and 1000. Forty-eight hours post-infection, the cell viability was determined by CCK-8 assay according to the manufacturer’s instructions. We added 10 µL of CCK-8 solution (WST-8 [2-(2-methoxy-4-nitrophenyl)-5-(2, 4-disulfophenyl)-2H-tetrazolium, monosodium salt]) to 100 µL of cell suspensions in 96-well plates. Absorbance values were recorded using a spectrophotometer (Tecan GmbH, Maennedorf, Switzerland) at 450 nm, with a reference wavelength of 600 nm 1–2 h after addition of the CCK-8 solution. The absorbance values of infected cells were normalized with controls and the percentage of cell proliferation at different MOI of *C. concisus* was calculated. 

### 4.9. Statistical Analysis

All data are expressed as the mean value ± standard error of the mean (SEM). Statistical analyses were performed with GraphPad Prism (GraphPad Software version 5.0, Inc., San Diego, CA, USA). Unpaired t-test was used to compare the mean values of the independent groups. To compare the data sets with unequal variances, we used unpaired t-test with Welch’s correction. *p* < 0.05 was considered statistically significant.

## Data Availability

No big data repositories needed. The raw data supporting the findings of this manuscript will be made available by the corresponding author, R.B., or the first author, P.K.N., to any qualified researcher upon reasonable request.

## Supplementary Materials

The following are available online at https://www.mdpi.com/1422-0067/22/4/2043/s1, Figure S1. Cytokines released from THP-1 cells in co-culture with cell monolayers. Figure S2. Cell viability in *Campylobacter concisus*-infected HT-29/B6-GR/MR cells and macrophage-like THP-1 cells 48 h post infection.

## Abbreviations

ATCC	American Type Culture Collection
BCA	Bicinchoninic acid
cAMP	Adenosine 3′,5′ -cyclic monophosphate
CBA	C Cytometric Bead Array
CCK-8	Cell counting kit-8
CD	Crohn’s Disease
CFU	Colony forming units
CLSM	Confocal laser scanning microscopy
CREB1	cAMP responsive element binding protein 1
CRP	C-Reactive Protein
CWD	Classical Whipple’s Disease
DAPI	4′-6-diamidino-2-phenylindole dihydrochloride
DBA	DexaDexamethasone, butyrate-sodium salt, and aldosterone
DSS	Dextran sodium sulfate
EDTA	Ethylenediaminetetraacetic acid
EGTA	Ethylene glycol-bis(β-aminoethyl ether)-N,N,N′,N′-tetraacetic acid
ENaC	Epithelial sodium channel
EPEC	Enteropathogenic *Escherichia coli*
ERK	Extracellular signal-regulated kinases
FACS	Fluorescence-Activated Cell Sorting
FCS	Fetal calf serum
FITC	Fluorescein isothiocyanate
GAPDH	Glyceraldehyde 3-phosphate dehydrogenase
GR	Glucocorticoid receptor
HIV	Human immunodeficiency virus
IBD	Inflammatory bowel disease
IFN	Interferon
IL	Interleukin
LPS	Lipopolysaccharide
MOI	Multiplicity of infection
MR	Mineralocorticoid receptor
mRNA	messengerRNA
NF-κB	Nuclear factor kappa light chain enhancer of activated B cells
p.i.	Post infection
PBMC	Peripheral blood mononuclear cells
PBS	Phosphate buffered saline
PCF	Polycarbonate membrane filters
PFA	Paraformaldehyde
PMA	phorbol 12-myristate 13-acetate
PVDF	Polyvinylidene fluoride
PVP	Polyvinylpyrrolidone
RNA-Seq	RNA sequencing
RPMI	Rosewell Park Memorial Institute
RT	Room temperature
SDS	Sodium dodecyl sulfate
T3SS	Type-III secretion system
T6SS	Type-VI secretion system
TBS	Tris-buffered saline
TER	Transepithelial electrical resistance
TJ	Tight junction
tTJ	Tricellular TJ
TNF	Tumor necrosis factor
TUNEL	Terminal deoxynucleotidyl transferase dUTP nick end labeling
UC	Ulcerative colitis
ZO-1	Zonula occludens protein-1
