# Supplementary material for: Immune-Mediated Aggravation of the Campylobacter concisus-Induced Epithelial Barrier Dysfunction
Source: Int J Mol Sci. 2021 Feb 19;22(4):2043. doi: 10.3390/ijms22042043 (PMC7922099; doi:10.3390/ijms22042043)
Supplement: Supplementary file 1 [file ijms-22-02043-s001.pdf]

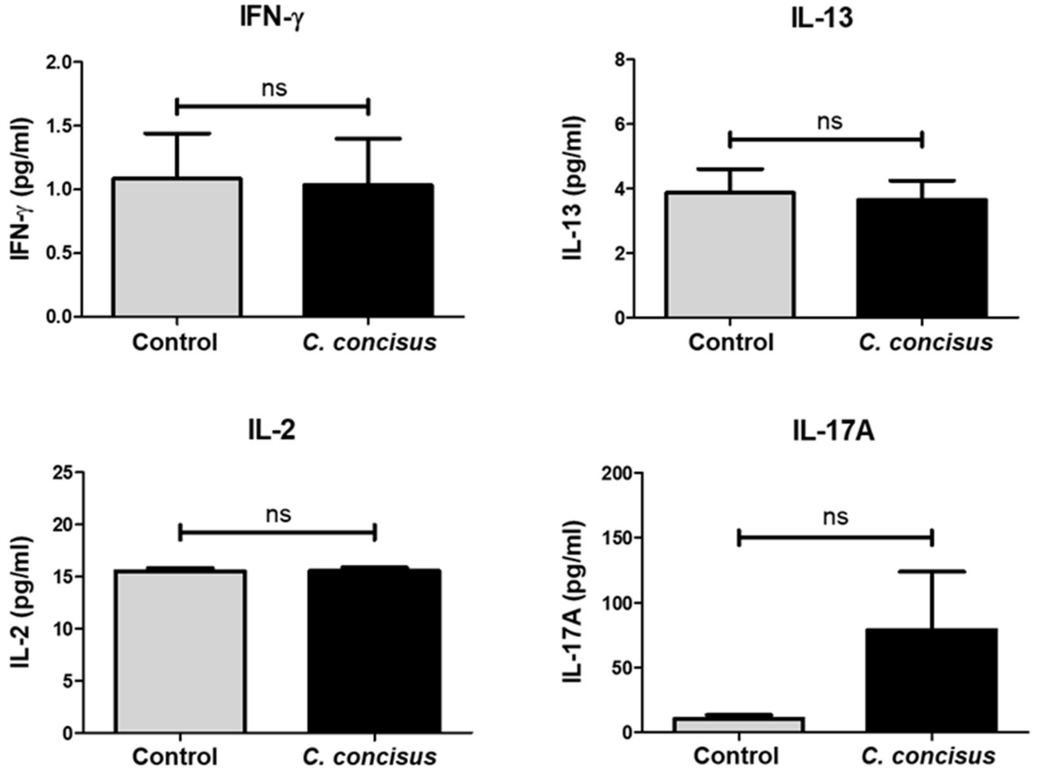

Figure S1. Cytokines released from THP-1 cells in co-culture with cell monolayers**.** Under co-culture condition (HT-29/B6-GR/MR cell monolayers with M1-macrophage-like THP-1 cells on the basal side), the cytokines released from differentiated THP-1 cells were quantified. The cytokines IFN-γ, IL-13, IL-2 and IL-17A released from the infected cells (black bars) were compared to cytokines released from uninfected controls (bright grey bars) (*n* = 6 in two independent experiments, *ns* = not significant). The cell monolayers were infected with *Campylobacter concisus* at MOI of 200 on both apical and basal side.

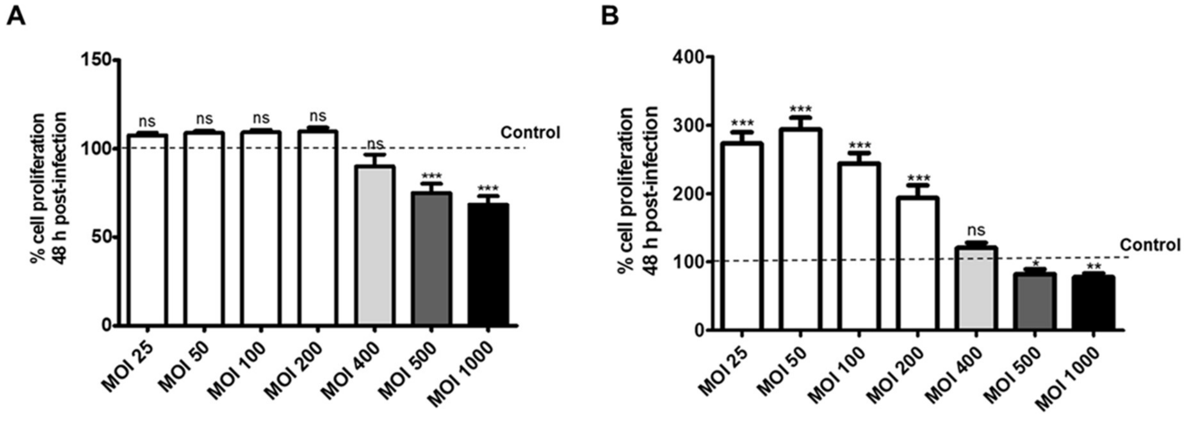

Figure S2. Cell viability in *Campylobacter concisus*-infected HT-29/B6-GR/MR cells and macrophage-like THP-1 cells 48 h post infection. Absorbance values recorded at 450 nm with a reference wavelength of 600 nm in controls and *C. concisus*-infected cells at different MOI ranging from 25 to 1000. Percentage of increase or decrease in cell proliferation was estimated after normalizing the absorbance values with controls. (**A**) HT-29/B6-GR/MR cells. (**B**) M1-macrophage-like THP-1 cells (WST-8 in the CCK8 assay; *n* = 4-17 in two to three independent experiments, *ns* = not significant, *** $p < 0.001$, ** $p < 0.01$, * $p < 0.05$).
